# Muscle-Related Parameters as Determinants of Survival in Patients with Stage I-III Gastric Cancer Undergoing Gastrectomy

**DOI:** 10.7150/jca.61199

**Published:** 2021-07-25

**Authors:** Soomin An, Wankyu Eo, Youn-Jung Kim

**Affiliations:** 1 College of Nursing, Hallym Polytechnic University, Gangwon-do, Republic of Korea.; 2 College of Medicine, Kyung Hee University, Seoul, Republic of Korea.; 3 College of Nursing Science, Kyung Hee University, Seoul, Republic of Korea.

**Keywords:** Gastrectomy, Stomach Neoplasms, Paraspinal Muscles, Muscle, Skeletal

## Abstract

**Purpose**: This study aimed to evaluate the prognostic potential of muscle-related parameters (MRPs) at the level of the third lumbar vertebra (L3) using computerized tomography (CT) images in patients with stage I-III gastric cancer (GC) who underwent curative gastric resection.

**Methods**: Patients with stage I-III GC who underwent curative gastric resection between October 2006 and June 2014 were enrolled in this study. In addition to demographic and clinical parameters, MRPs, such as skeletal muscle index (SMI), skeletal muscle radiation attenuation (SMRA), paraspinal muscle index (PMI), and paraspinal muscle radiation attenuation (PMRA), at the L3 level using CT images were collected and analyzed. The Kaplan-Meier method was used to estimate survival, and a Cox proportional hazard model was used to calculate the hazard ratio. In addition, the Pearson correlation coefficient was obtained as a measure of the linear relationship between the variables.

**Results**: Data from 339 patients (233 men and 116 women) were analyzed. A strong correlation between SMI and PMI (*r* = 0.91); and SMRA and PMRA (*r* = 0.80) were observed, but only weak correlations between SMI and SMRA; and PMI and PMRA were found. When using the Cox model, SMRA or PMRA was a determinant of survival, but SMI and PMI were not. In the full model formed by adding SMRA or PMRA to a baseline model that included demographic and clinical variables, the C-index increased above 0.8, indicating excellent discrimination for both overall survival (OS) and disease-free survival (DFS). Moreover, the C-index of the model containing PMRA was higher than that of the model containing SMRA. Finally, there was a weak correlation between the prognostic nutritional index and SMRA or PMRA.

**Conclusions**: With the multivariate Cox model, SMRA and PMRA appeared to determine survival. In addition, adding SMRA or PMRA to the baseline model increased the C-index above 0.8, indicating excellent discrimination for both OS and DFS. Moreover, compared to SMRA, the model containing PMRA appears to be a more accurate model for survival determination.

## Introduction

Although surgical resection is the most effective treatment for potentially curable gastric cancer (GC), radical gastrectomy tends to be associated with high morbidity and mortality. In addition, around 20% of patients who undergo gastrectomy experience recurrence, which may translate into poor survival rates 1. Therefore, the establishment of biomarkers that can predict postoperative complications, recurrence, and long-term survival is warranted. However, biomarkers that have been developed to date are still unsatisfactory 2.

Recently, computerized tomography (CT) has been considered the gold standard for evaluating muscle mass and quality due to its accuracy, operator independence, reproducibility, and non-invasiveness. As CT scans are a regular part of the standard cancer staging in most cases, additional exposure to radiation doses can be avoided for body composition measurement in cancer patients 3. CT-based measurement of the skeletal muscle area (SMA) is usually performed in the total abdominal wall musculature (i.e., erector spinae, multifidus, quadratus lumborum, psoas, rectus abdominis, external obliques, internal obliques, and transversus abdominis). Skeletal muscle index (SMI), an SMA adjusted for height squared at the third lumbar vertebra (L3) level, is considered a major determinant of muscle mass. Skeletal muscle index below the threshold has been considered a risk factor for survival outcomes in patients with gastrointestinal malignancies 4-10. Fat in skeletal muscles is present in the form of intermuscular adipose tissue, intramuscular adipose tissue, or intramyocellular lipids 11. CT can detect changes in muscle fat while circumventing the need for invasive muscle biopsy 12. Muscle radiation attenuation (MRA), which is calculated using CT images, is a radiologic index of muscle fat content; the values of the mean MRA are inversely correlated with muscle fat content 12. Skeletal muscle radiation attenuation (SMRA) within the total abdominal wall musculature has been reported as a determinant of survival in various gastrointestinal tumors 13-16.

Recently, CT-based measurements of the paraspinal muscle area (PMA) have attracted attention. The paraspinal muscle index (PMI), a PMA adjusted for height squared at the L3 level, has been reported to be a significant determinant of survival in patients with gastrointestinal malignancies 16-18. In addition, paraspinal muscle radiation attenuation (PMRA) was considered a significant prognostic factor for survival outcomes in stage I-II GC patients 19. However, the clinical significance of PMI and PMRA compared to that of SMI and SMRA is unclear.

Therefore, the purpose of the present study was to compare the clinical significance of four muscle-related parameters (i.e., SMI, SMRA, PMI, and PMRA) as prognostic factors for survival, using L3 level preoperative CT imaging in a cohort of stage I to III GC patients.

## Methods

### Patients

Patients who underwent gastric resection for GC between June 2006 and October 2014 at a single university hospital were retrospectively evaluated. The inclusion criteria were as follows: patients with/who (i) primary GC, according to Lauren's histological classification 20; (ii) stage I through III, according to the 7th edition of the American Joint Committee on Cancer Staging 21; (iii) underwent R0 resection; and (iv) underwent gastrectomy by expert gastroenterologists who have participated in more than 50 GC resections annually.

The exclusion criteria were: patients (i) with previous malignancies within the last 5 years, or concurrent second malignancies; (ii) who received neoadjuvant chemotherapy, radiation therapy, or other anti-cancer treatment prior to surgery; (iii) with stage IV; (iv) absence of R0 resection; (v) with a history of positive tests for human immunodeficiency virus, severe infection within 4 weeks prior to surgery, or active autoimmune diseases that require systemic or immunosuppressive agents; (vi) with chronic kidney disease (stage 4 or 5); (vii) with chronic obstructive pulmonary disease; (viii) who underwent surgical interventions for lumbar spine disorders 22; (ix) without Korean resident registration number; and (x) without a preoperative abdominal CT scan that could be analyzed.

### Baseline clinical characteristics

Demographic data (e.g., age, sex, height, body weight, and body mass index) and pathological parameters (e.g., tumor location, tumor size, type of gastrectomy, stage, Lauren's histological classification, and lymphatic, vascular, and perineural invasion) were collected and analyzed.

Selected blood tests included peripheral blood leukocyte count, hemoglobin concentration, platelet count, and serum albumin level. Blood test results were analyzed using tests performed within seven days before surgery. If more than one test result was available, the test result closest to the date of surgery was selected for further analysis. The diagnosis of anemia was based on hemoglobin concentrations below 13 g/dL in men and 12 g/dL in women. Blood test results were also used to calculate the prognostic nutritional index (PNI), 10 × serum albumin level (g/L) + 0.005 × absolute lymphocyte count (ALC) 23.

### Muscle-related parameters

Only CT images taken as part of a routine staging work-up within 30 days prior to surgery were analyzed. After identification of the landmark at the L3 level, the corresponding single, axial CT image was extracted by a musculoskeletal radiologist and saved as a DICOM image file 24. In this study, the slice thickness was set to 5 mm. The image was analyzed by a trained nurse using ImageJ^®^ v.1.37, a Java-based image analysis program, which went through an inspection process by a physician with expertise in the musculoskeletal system.

**Figure 1:**
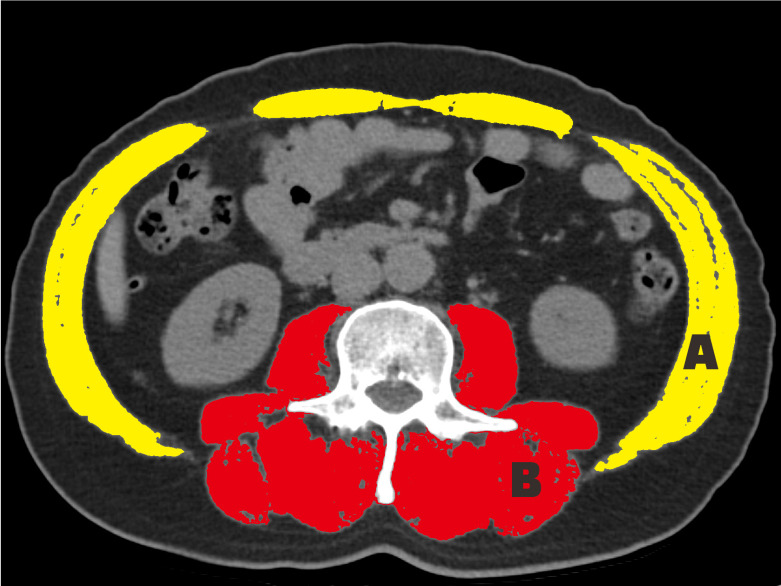
Cross-sectional area at the level of L3. The skeletal muscle index and skeletal muscle radiation attenuation are measured in areas A and B; the paraspinal muscle index and paraspinal muscle radiation attenuation are measured in area B.

To measure the SMA, the total abdominal wall musculature was selected. The paraspinal muscles (i.e., the erector spinae, multifidus, quadratus lumborum, and psoas muscles) were selected for measurement (Fig. 1). The SMA and PMA were adjusted by the square of the height, which produced the SMI and PMI, respectively 25. To measure the muscle-related parameters (MRP), a Hounsfield unit (HU) threshold range of -29 to +150 was applied.

### Statistical analysis

Clinical features are described as median and interquartile range. The Mann-Whitney U test or Chi-squared test was used for intergroup comparisons, depending on the nature of the variables. Variables without well-known cutoff points were analyzed as continuous variables without an intentional dichotomy. Disease-free survival (DFS) was defined as the interval from the date of GC surgery to the date of recurrence or death from any cause, whichever came first. Overall survival (OS) was defined as the interval from the date of GC surgery to the date of death from any cause. Patients who did not experience cancer recurrence or death from any cause were censored at the last follow-up.

The survival rate was estimated using the Kaplan-Meier method, and the statistical significance between survival curves was tested using the log-rank test. Statistical significance was set at *P <*0.05. In addition, the Cox proportional hazards model was used to calculate hazard ratios, which were performed only on variables that met the proportional hazards assumption on the basis of graphic plots of Schoenfeld residuals. Only variables with *p* <0.05, in the univariate analysis, were included in the multivariate Cox model.

All statistical analyses were performed using R packages. Variance inflation factors (VIF) were calculated for the diagnosis of multicollinearity of the variables. To measure the model's discriminative capacity, the concordance index (C-index) for the Cox model was used. In addition, a bootstrap cross-validation estimate of the C-index at different time points was applied to assess and compare the discriminative power of the two regression models (i.e., baseline vs. full model). The number of bootstrap samples was 1000, and sampling with replacement from the original dataset was accomplished.

Finally, Pearson correlation coefficients were obtained as a measure of the linear relationship between MRPs and the linear relationship between PNI and MRPs.

## Results

### Demographic and clinical characteristics of patients

The baseline demographic and clinical characteristics of the patients are presented in Table 1. The median age of the patients was 60 years, with more men (65.8%) than women. There were 207 (61.1%) patients in stage I, 66 (19.5%) in stage II, and 66 (19.5%) in stage III. Sixty-eight patients (20.1%) underwent total gastrectomy. The intestinal type, according to Lauren's classification, was the most common (51.9%), and perineural invasion was found in 24 (7.1%) patients. Anemia was diagnosed in 122 (36.0%) patients, while 21 (6.2%) had hypoalbuminemia with serum albumin levels less than 3.5 g/dL.

### Impact of muscle-related parameters on the Kaplan-Meier curves

Because there were significant differences in the medians of the MRPs (i.e., SMI, SMRA, PMI, and PMRA) between sexes (*p*<0.001 for all variables), the MRPs were dichotomized for each sex, using thresholds determined using the receiver operating characteristic curve analysis (Table 2). The number of patients below the SMI and SMRA thresholds was 143 (42.2%) and 111 (32.7%), respectively. In addition, 178 (52.5%) and 137 (40.4%) patients were below the PMI and PMRA thresholds, respectively.

**Figure 2:**
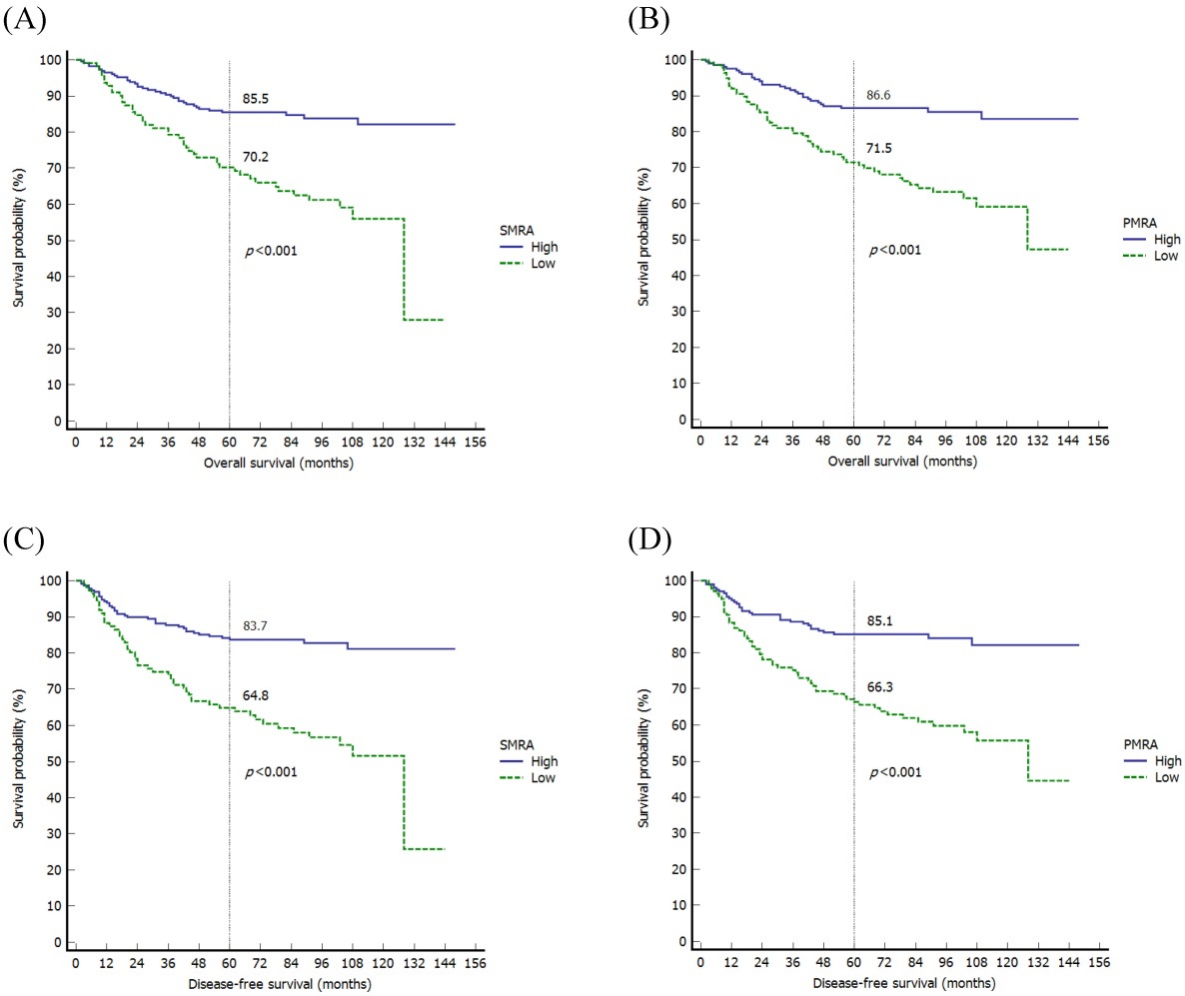
Kaplan-Meier survival curves of the overall survival according to the SMRA (A) and PMRA (B), and disease-free survival according to the SMRA (C) and PMRA (D). SMRA, skeletal muscle radiation attenuation; PMRA, paraspinal muscle radiation attenuation

When evaluating the MRPs using the Kaplan-Meier method, there were significant differences in OS among variables, such as SMRA (*p*<0.001), PMI (*p*=0.027), and PMRA (*p*<0.001). As for DFS, there were significant differences in DFS in variables such as SMRA (*p*<0.001) and PMRA (*p*<0.001) (Fig. 2).

### Cox model of the risk factors for OS

Significant variables in the univariate Cox proportional hazard model for OS were older age, tumor size, tumor stage, lymphatic invasion, vascular invasion, perineural invasion, anemia, PNI, SMRA, PMI, and PMRA (Table 3). Because there was a strong correlation between SMRA and PMRA (*r*=0.80), these two variables were analyzed separately by adding SMRA or PMRA to baseline models. Therefore, Model 1 included SMRA in addition to demographic and clinical variables, and model 2 included PMRA in addition to demographic and clinical variables.

**Figure 3:**
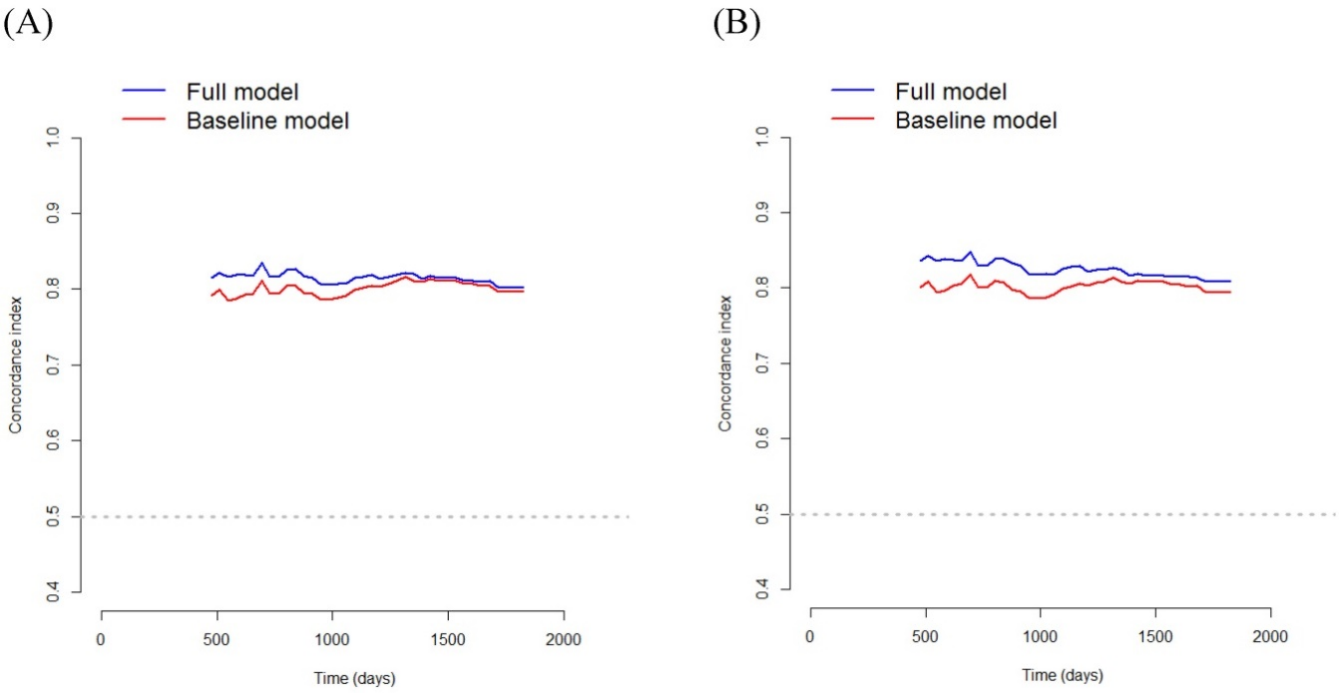
Bootstrap cross-validation estimates of the C-index for overall survival at different time points to assess and compare the discriminative power of two regression models; baseline and full models. On panel A, the baseline model includes 4 variables (i.e., older age, stage, perineural invasion, and PNI), and the full model includes SMRA in addition to the variables in the baseline model; and on panel B, the baseline model includes 5 variables (i.e., older age, stage, perineural invasion, anemia, and PNI), and the full model includes PMRA in addition to the variables in the baseline model. PNI, prognostic nutritional index; SMRA, skeletal muscle radiation attenuation; PMRA, paraspinal muscle radiation attenuation.

In Model 1, multivariate survival analysis revealed that older age (hazard ratio [HR] 2.07, *p=*0.002), stage (HR 3.53, 95% CI 2.20-5.65, *p<*0.001), perineural invasion (HR 2.60, *p=*0.005), PNI (HR 0.91, *p<*0.001), and SMRA (HR 1.82, *p*=0.012) were significant prognostic factors for OS (Table 3). The VIFs for older age, stage, perineural invasion, PNI, and SMRA were 1.10, 1.12, 1.14, 1.12, and 1.12, respectively. The C-index was 0.797 in the baseline model (i.e., older age, stage, perineural invasion, and PNI), and it was 0.806 in the full model (i.e., SMRA in addition to the baseline model). Bootstrap cross-validation also revealed a higher C-index for the full model than that for the baseline model (Table 3, Fig. 3).

In Model 2, multivariate survival analysis revealed the following significant variables: older age (HR 2.10, *p=*0.002), stage (HR 3.35, *p<*0.001), perineural invasion (HR 2.66, *p=*0.004), anemia (HR 1.69, *p=*0.039), PNI (HR 0.93, *p<*0.001), and PMRA (HR 2.22, *p*< 0.001) (Table 3). The VIFs for older age, stage, perineural invasion, anemia, PNI, and PMRA were 1.12, 1.15, 1.17, 1.21, 1.25, and 1.06, respectively. The C-index was 0.799 in the baseline model (i.e., older age, stage, perineural invasion, anemia, and PNI), and 0.818 in the full model (i.e., PMRA in addition to the baseline model). Bootstrap cross-validation also revealed a higher C-index for the full model than that for the baseline model (Table 3, Fig. 3).

### Cox model of the risk factors for DFS

Except for PMI, the same variables found to be significant in the OS analysis using the univariate Cox model were also found to be significant in the DFS analysis (Table 4).

**Figure 4:**
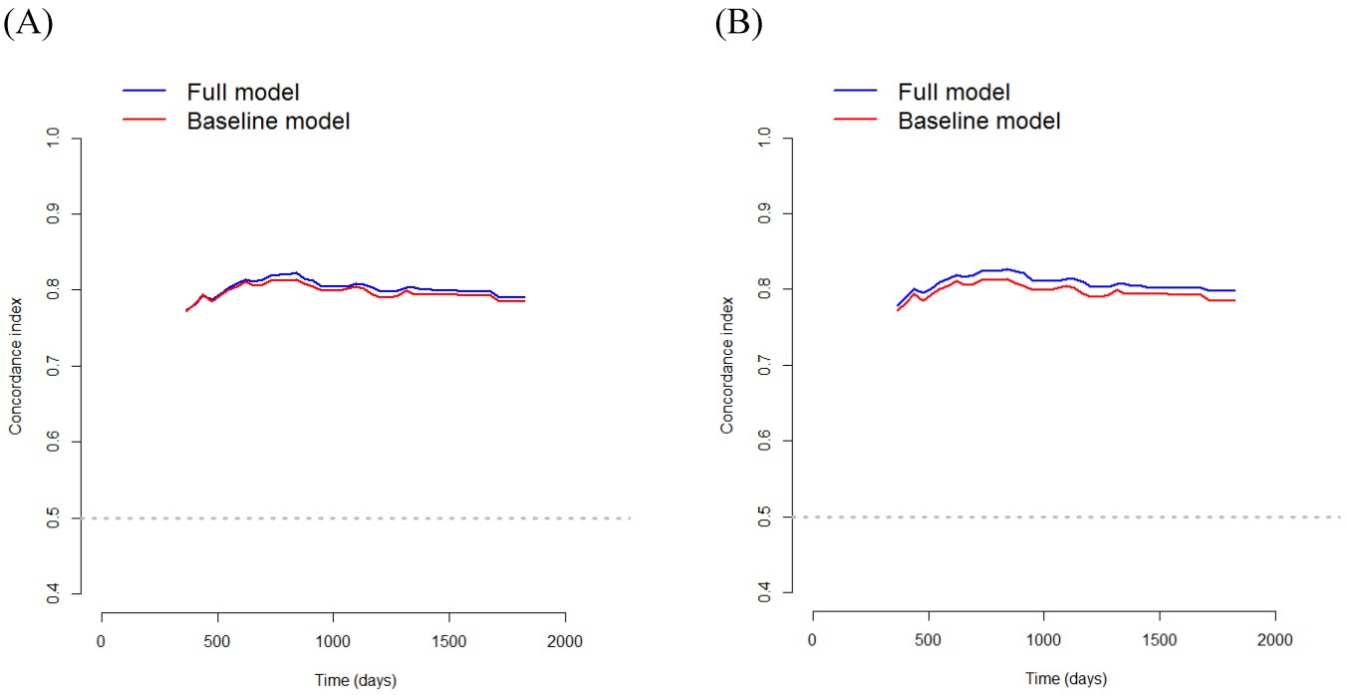
Bootstrap cross-validation estimates of the C-index for disease-free survival at different time points to assess and compare the discriminative power of two regression models; baseline and full models. On panel A, the baseline model includes 4 variables (i.e., older age, stage, perineural invasion, and PNI), and the full model includes SMRA in addition to variables in the baseline model; and on panel B, the baseline model includes 4 variables (i.e., older age, stage, perineural invasion, and PNI), and the full model includes PMRA in addition to variables in the baseline model. PNI, prognostic nutritional index; SMRA, skeletal muscle radiation attenuation; PMRA, paraspinal muscle radiation attenuation.

In Model 1, multivariate survival analysis revealed that older age (HR 1.80, *p=*0.010), stage (HR 4.10, *p<* 0.001), perineural invasion (HR 2.20, *p=*0.018), PNI (HR 0.91, *p<*0.001), and SMRA (HR 1.91, *p=*0.005) were significant prognostic factors for DFS (Table 4). The VIFs for older age, stage, perineural invasion, PNI, and SMRA were 1.11, 1.09, 1.14, 1.12, and 1.14, respectively. The C-index was 0.787 in the baseline model (i.e., older age, stage, perineural invasion, and PNI), and it was 0.802 in the full model (i.e., SMRA in addition to baseline model). Bootstrap cross-validation also revealed a higher C-index for the full model than for the baseline model (Table 4, Fig. 4).

In Model 2, multivariate survival analysis revealed the following significant variables: older age (HR 1.82, *p=*0.009), stage (HR 4.20, *p<* 0.001), perineural invasion (HR 2.15, *p=*0.021), PNI (HR 0.91, *p<*0.001), and PMRA (HR 2.18, *p<*0.001) (Table 4). The VIFs for older age, stage, perineural invasion, PNI, and PMRA were 1.11, 1.09, 1.12, 1.10, and 1.05, respectively. The C-index was 0.787 in the baseline model (i.e., older age, stage, perineural invasion, and PNI), and it was 0.809 in the full model (i.e., PMRA in addition to baseline model). Bootstrap cross-validation also revealed a higher C-index for the full model than that for the baseline model (Table 4, Fig. 4).

### Pearson correlation coefficients as a measure of the linear relationship

**Figure 5:**
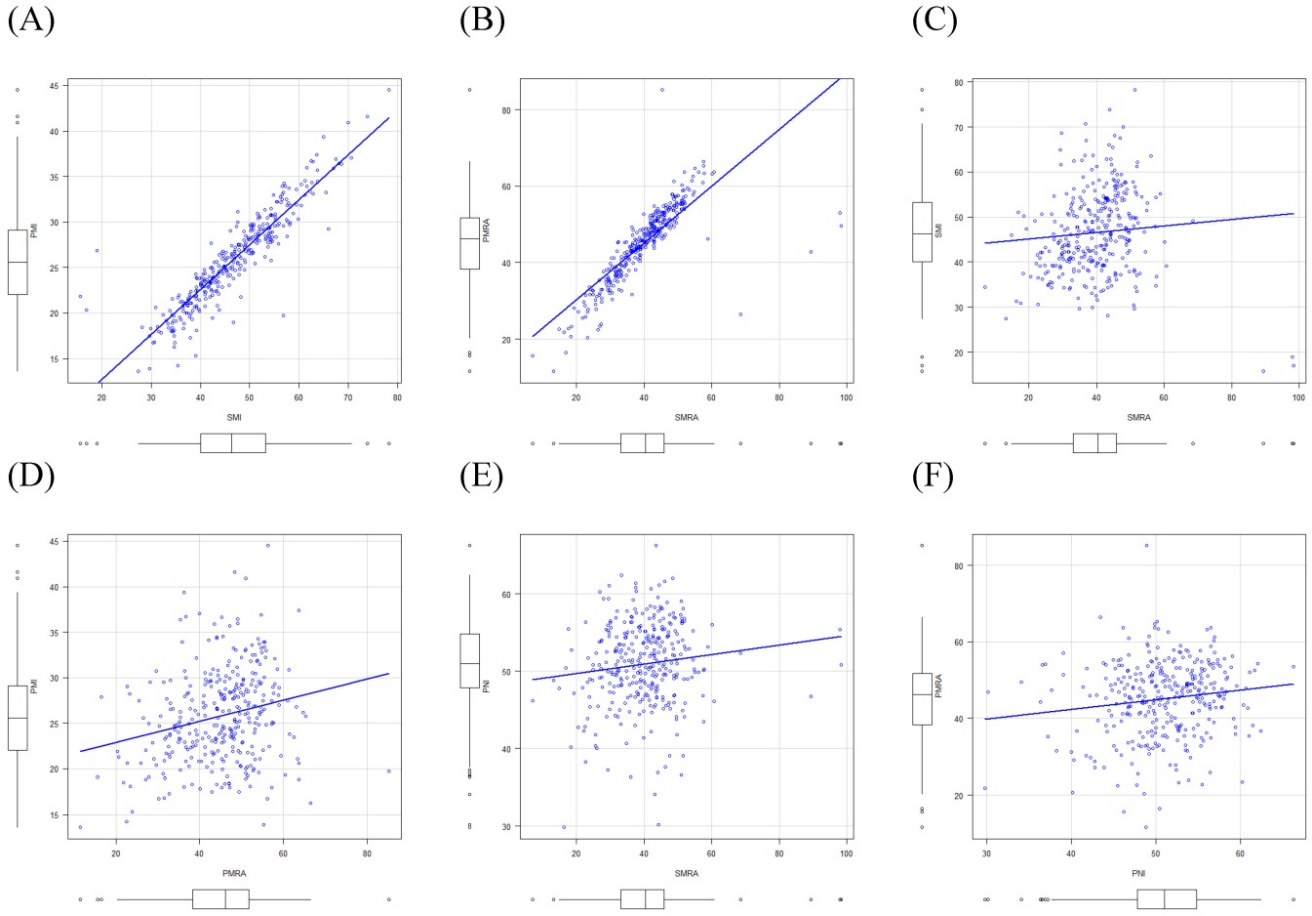
Correlations between muscle-related parameters, and between the prognostic nutritional index and muscle-related parameters. SMI, skeletal muscle index; PMI, paraspinal muscle index; SMRA, skeletal muscle radiation attenuation; PMRA, paraspinal muscle radiation attenuation; PNI, prognostic nutritional index.

There were strong correlations between SMI and PMI (*r*=0.91) and between SMRA and PMRA (*r*=0.80). However, there were only weak correlations between SMI and SMRA (*r*=0.08) and between PMI and PMRA (*r*=0.23). Moreover, weak correlations were found between PNI and MRPs, such as SMI, SMRA, PMI, and PMRA (*r*=0.17, *r*=0.12, *r*=0.20, and *r*=0.14, respectively) using Pearson's correlation tests (Fig. 5).

## Discussion

The purpose of the current study was to compare the clinical significance of MRPs as prognostic factors for survival, using L3 level preoperative CT imaging in a cohort of stage I to III GC patients. In this study, SMRA or PMRA appeared to be significant variables in determining OS and DFS. Adding SMRA or PMRA to the baseline model increased the C-index above 0.8, indicating excellent discrimination for both OS and DFS. Moreover, models that include PMRA rather than SMRA appear to be more accurate survival determinants in GC.

In this study, we calculated the SMI, the SMA normalized for height squared, for use as an indicator of muscle mass. Most commonly used SMI-based definitions for low muscle mass include the international definition and Martin's definition 26. While the international definition was reported as <55 cm^2^/m^2^ for men and <39 cm^2^/m^2^ for women 27, Martin's definition was <43 cm^2^/m^2^ for men with a BMI<25, <53 cm^2^/m^2^ for men with a BMI >25, and <41 cm^2^/m^2^ for women 14. Although several studies have been conducted according to Martin's definition, the main concern is that male thresholds can cause confusion. Therefore, a new standardized definition should be established taking into account the inconsistency of the definition of low muscle mass currently in use 26. In this study, we determined the cutoff values based on our patient cohort. As the SMI was sex-dependent, it was dichotomized using sex-specific cutoffs. In the present study, SMI was not a significant determinant of OS or DFS when using the Cox model. The results of this study are compatible with those of previous studies on gastrointestinal tumors 9, 16. In addition to the SMI, we measured the PMI and PMA normalized for height squared for statistical analysis. As PMI was sex-dependent, it was dichotomized using sex-specific cutoffs. In the present study, using the multivariate Cox model, PMI was not an important determinant of OS or DFS. The results of this study are in line with those of a previous study on gastrointestinal tumors 16. Therefore, the two indicators of muscle mass (i.e., SMI and PMI) were not determinants of survival using a multivariate Cox model. However, since only a limited number of studies have been conducted in the field, further research is needed to reach a consensus.

In this study, SMRA was dichotomized using sex-specific cutoffs and adjustment for age was performed by including age as a variable at entry 28. When applying the multivariate Cox model (model 1), SMRA was a significant determinant of OS (HR 1.82, *p*=0.012) and DFS (HR 1.91, *p*=0.005). The role of SMRA as a significant prognostic factor for survival in patients with GC has been reported in several studies 13, 14, 16. PMRA was also dichotomized with sex-specific cutoffs, and age adjustment was performed by including age as a variable at entry. Using the multivariate Cox model (model 2), PMRA was a significant determinant of OS (HR, 2.22; *p*<0.001) and DFS (HR, 2.18; *p*<0.001). The results of this study are compatible with those of previous studies on gastrointestinal tumors 18, 19. Therefore, both SMRA and PMRA are suggested to be significant determinants of OS and DFS.

In the present study, while SMRA or PMRA was an important determinant of OS and DFS, SMI or PMI was not a determinant of survival. The results of this study are compatible with those of previous studies on gastrointestinal tumors 16, 18. Thus, muscle quality (i.e., SMRA and PMRA) as compared to muscle mass (i.e., SMI and PMI) appears to be a significant determinant of survival. In addition, as there was no significant correlation between SMI and SMRA, and between PMI and PMRA, muscle quality and muscle mass appeared to be mutually exclusive.

Using the baseline multivariate Cox model for OS, the C-index was 0.797 for model 1 and 0.799 for model 2. However, by using the full multivariate Cox model containing MRAs, the C-index increased up to 0.806 for model 1 and 0.818 for model 2, indicating excellent discrimination. In addition, using the baseline multivariate Cox model for DFS, the C-index was 0.787 for model 1 and 0.787 for model 2. However, using the full multivariate Cox model containing MRAs, the C-index increased to 0.802 for model 1 and 0.810 for model 2, indicating excellent discrimination. Therefore, the results of this study highlight the clinical significance of MRA in this model. Moreover, the C-index of the full multivariate Cox model for OS was 0.806 for model 1 and 0.818 for model 2, while the C-index of the full multivariate Cox model for DFS was 0.802 for model 1 and 0.810 for model 2. Thus, Model 2 appears to be more accurate than Model 1 in predicting OS and DFS.

While SMRA and PMRA were significant determinants for OS and DFS, the PNI, an indicator of nutritional status, was also an important determinant in the multivariate Cox model. Nutritional status (i.e., PNI), and muscle quality (i.e., SMRA and PMRA) appeared to be mutually exclusive as there was no multicollinearity between PNI and SMRA or PMRA in these variables according to the test for VIFs, and no significant correlation between PNI and SMRA or PMRA using the Pearson correlation test.

The strengths of our study are as follows: First, with a multivariate Cox model, SMI or PMI was not a determining factor for survival. However, SMRA or PMRA could predict OS and DFS, highlighting the importance of skeletal muscle quality (i.e., SMRA or PMRA) as determinants of survival. Moreover, we found no significant correlation between PNI and SMRA or PMRA. Therefore, muscle quality appears to be independent of the patient's nutritional status (i.e., PNI). Second, using the full model with the addition of SMRA or PMRA to the baseline model increased the C-index above 0.8, indicating the model's excellent discrimination for both OS and DFS. Additionally, models with PMRA appear to be more accurate survival determinants because models with PMRA have a higher C-index than those in models with SMRA.

However, since this study had several limitations, the results of the study should be interpreted carefully. First, this study was performed retrospectively; therefore, omission of data including CT images was inevitable and may have affected the results. Second, although random errors and potential biases were controlled from the study design to implementation, the lack of external validation was another limitation of our study. Third, since this was a retrospective study, there was no opportunity to provide special interventions to improve postoperative outcomes in patients with PMRA below the threshold. Finally, the cutoffs for the dichotomization of MRPs in our cohort were determined using receiver operating characteristic curve analysis. As such, the cutoffs presented in this study may not be applicable to patients with malignant tumors other than GC.

In conclusion, unlike skeletal muscle mass (i.e., SMI or PMI) in GC patients, skeletal muscle quality (i.e., SMRA or PMRA) appears to be an important determinant of survival, and muscle quantity and quality appear to be mutually exclusive. There was a strong correlation between SMI and PMI and between SMRA and PMRA; therefore, the clinical role of the non-paraspinal muscle in the abdomen appears to be negligible. Adding SMRA or PMRA to the baseline model increased the C-index above 0.8, indicating excellent differentiation for both OS and DFS. Compared to the model with SMRA, the model with PMRA appears to be a more accurate survival determination model. Finally, there was no significant correlation between PNI and SMRA or PMRA. Therefore, the prognostic importance of SMRA and PMRA appears to be independent of the patient's nutritional status. Since PMRA is a newly characterized determinant of survival in GC, its prognostic importance requires further validation prior to clinical application. In addition, measurements of muscle mass and MRAs using CT require standardization for comparison between studies.

## Figures and Tables

**Table 1:** Characteristics of patients

Variables	Median (IQR): or *n* (%)
**Age (years)**	60.0 (52.0-69.0)
**Sex**	
Male	223 (65.8%)
Female	116 (34.2%)
**BMI (kg/m^2^)**	23.7 (21.4-25.8)
**Site of tumor**	
Upper	32 (9.4%)
Middle	139 (41.0%)
Lower	164 (48.4%)
Diffuse	4 (1.2%)
**Size of tumor (cm)**	3.0 (2.0-5.2)
**Nodal invasion**	
No	223 (65.8%)
Yes	116 (34.2%)
**Stage**	
I	207 (61.1%)
II	66 (19.5%)
III	66 (19.5%)
**Gastrectomy**	
Partial	271 (79.9%)
Total	68 (20.1%)
**Histology** †	
Intestinal	176 (51.9%)
Diffuse	81 (23.9%)
Mixed	68 (20.1%)
Unknown	14 (4.1%)
**Lymphatic invasion**	
No	231 (68.1%)
Yes	108 (31.9%)
**Vascular invasion**	
No	322 (95.0%)
Yes	17 (5.0%)
**Perineural invasion**	
No	315 (92.9%)
Yes	24 (7.1%)
**WBC (per μL)**	6500 (5400-8000)
**Anemia** ‡	
No	217 (64.0%)
Yes	122 (36.0%)
**Platelet (10^3^/μL)**	236 (203-278)
**Serum albumin (g/dL)**	4.1 (3.9-4.3)
**PNI**	51.0 (47.8-54.8)
**Adjuvant chemotherapy**	
No	215 (63.4%)
Yes	124 (36.6%)

† Lauren classification ‡The cutoff points were 12 g/dL in female patients and 13 g/dL in male patients. BMI, body mass index; WBC, white blood cell; PNI, prognostic nutritional index

**Table 2:** Medians and threshold values of muscle-related parameters according to sex

	Median (IQR)			Threshold values†	
	Male (*n* = 223)	Female (*n* = 116)	*p*-value	Male (*n* = 223)	Female (*n* = 116)
SMI (cm^2^/m^2^)	50.2 (44.8-55.1)	39.2 (36.3-42.4)	<0.001	46.48	40.77
SMRA (HU)	42.5 (35.4-47.6)	37.3 (30.1-42.7)	<0.001	40.56	26.39
PMI (cm^2^/m^2^)	27.6 (24.8-30.2)	22.2 (19.9-24.0)	<0.001	26.78	23.80
PMRA (HU)	48.1 (41.2-53.3)	43.7 (35.4-49.3)	<0.001	48.12	34.24

† Threshold was determined using the receiver operating characteristic curve. IQR, interquartile range; SMI, skeletal muscle index; SMRA, skeletal muscle radiation attenuation; PMI, paraspinal muscle index; PMRA, paraspinal muscle radiation attenuation

**Table 3:** Univariate Cox proportional hazards regression analysis of overall survival

Covariates	Univariate analysis		Multivariate analysis (Model 1)		Multivariate analysis (Model 2)	
	HR (95% CI)	*p*-value	HR (95% CI)	*p*-value	HR (95% CI)	*p*-value
Older age (yes vs. no) †	2.15 (1.59-3.88)	<0.001	2.07 (1.29-3.30)	0.002	2.10 (1.31-3.37)	0.002
Sex (female vs. male)	0.78 (0.49-1.26)	0.316				
BMI (kg/m^2^)	0.97 (0.90-1.04)	0.329				
Size of tumor (cm)	1.17 (1.12-1.23)	<0.001				
Stage (III vs. I/II)	5.38 (3.46-8.37)	<0.001	3.53 (2.20-5.65)	<0.001	3.35 (2.08-5.39)	<0.001
Histology (intestinal vs. others) ‡	0.91 (0.59-1.41)	0.678				
Lymphatic invasion (yes vs. no)	3.29 (2.11-5.13)	<0.001				
Vascular invasion (yes vs. no)	3.34 (1.67-6.69)	0.007				
Perineural invasion (yes vs. no)	2.76 (1.49-5.11)	0.002	2.60 (1.34-5.03)	0.005	2.66 (1.36-5.20)	0.004
Anemia (yes vs. no) §	3.10 (1.98-4.84)	<0.001			1.69 (1.03-2.77)	0.039
PNI	0.87 (0.84-0.91)	<0.001	0.91 (0.88-0.95)	<0.001	0.93 (0.89-0.97)	<0.001
SMI (low vs. high)	1.42 (0.92-2.20)	0.117				
SMRA (low vs. high)	2.75 (1.77-4.28)	<0.001	1.82 (1.14-2.91)	<0.012		
PMI (low vs. high)	1.66 (1.05-2.62)	0.029				
PMRA (low vs. high)	2.81 (1.78-4.44)	<0.001			2.22 (1.38-3.55)	<0.001

The concordance statistics for the full multivariate Cox model were 0.806 for model 1 and 0.818 for model 2. † The cutoff point is 65 years; ‡ Lauren classification; § The cutoff points are 12 g/dL in female patients and 13 g/dL in male patients. HR, hazard ratio; CI, confidence interval; BMI, body mass index; PNI, prognostic nutritional index; SMI, skeletal muscle index; SMRA, skeletal muscle radiation attenuation; PMI, paraspinal muscle index; PMRA, paraspinal muscle radiation attenuation.

**Table 4:** Multivariate Cox proportional hazards regression analysis of disease-free survival

Covariates	Univariate analysis		Multivariate analysis (Model 1)		Multivariate analysis (Model 2)	
	HR (95% CI)	*p*-value	HR (95% CI)	*p*-value	HR (95% CI)	*p*-value
Older age (yes vs. no) †	2.33 (1.53-3.56)	<0.001	1.80 (1.15-2.81)	0.010	1.82 (1.17-2.84)	0.009
Sex (female vs. male)	0.67 (0.42-1.08)	0.100				
BMI (kg/m^2^)	0.97 (0.91-1.03)	0.339				
Size of tumor (cm)	1.17 (1.12-1.23)	<0.001				
Stage (III vs. I/II)	5.88 (3.86-8.97)	<0.001	4.10 (2.63-6.40)	<0.001	4.20 (2.69-6.56)	<0.001
Histology (intestinal vs. others) ‡	0.91 (0.60-1.38)	0.651				
Lymphatic invasion (yes vs. no)	3.16 (2.07-4.81)	<0.001				
Vascular invasion (yes vs. no)	3.74 (1.93-7.24)	<0.001				
Perineural invasion (yes vs. no)	2.51 (1.36-4.62)	0.032	2.20 (1.14-4.23)	0.018	2.15 (1.12-4.11)	0.021
Anemia (yes vs. no) §	2.87 (1.88-4.37)	<0.001				
PNI	0.87 (0.84-0.90)	<0.001	0.91 (0.88-0.95)	<0.001	0.91 (0.88-0.95)	<0.001
SMI (low vs. high)	1.27 (0.83-1.92)	0.271				
SMRA (low vs. high)	2.90 (1.90-4.42)	<0.001	1.91 (1.21-2.99)	0.005		
PMI (low vs. high)	1.39 (0.91-2.13)	0.130				
PMRA (low vs. high)	2.81 (1.85-4.41)	<0.001			2.18 (1.40-3.41)	<0.001

The concordance statistics for the full multivariate Cox model were 0.802 for model 1 and 0.809 for model 2. † The cutoff point is 65 years; ‡ Lauren classification; § The cutoff points are 12 g/dL in female patients and 13 g/dL in male patients. HR, hazard ratio; CI, confidence interval; BMI, body mass index; PNI, prognostic nutritional index; SMI, skeletal muscle index; SMRA, skeletal muscle radiation attenuation; PMI, paraspinal muscle index; PMRA, paraspinal muscle radiation attenuation.
